# Multimodality imaging approach in cardiac asymptomatic subjects with systemic sclerosis followed at a tertiary center

**DOI:** 10.3389/fcvm.2026.1935675

**Published:** 2026-08-21

**Authors:** Riccardo Scagliola, Gian Marco Rosa, Sara Seitun, Francesco Puppo, Claudio Brunelli

**Affiliations:** 1 Cardiology Division, Cardinal G. Massaia Hospital, Asti, Italy; 2 Cardiovascular Disease Unit, University of Genoa, Genoa, Italy; 3 Radiology Division, San Martino Hospital, Genoa, Italy; 4 Department of Internal Medicine, University of Genoa, Genoa, Italy

**Keywords:** cardiac magnetic resonance, Doppler echocardiography, multimodality imaging, subtle cardiac impairment, systemic sclerosis

## Abstract

**Objective:**

To investigate the role of a multimodality imaging approach in detecting subclinical cardiac impairment in systemic sclerosis (SSc) patients.

**Methods:**

Between 2017 and 2019 we retrospectively collected data related to 50 consecutive SSc patients who underwent Doppler echocardiography and cardiac magnetic resonance (CMR). The data was compared with 50 controls matched for age, sex and cardiovascular risk predictors.

**Results:**

CMR revealed late gadolinium enhancement (LGE) (30% vs. 8%, *p* = 0.005), myocardial edema (14% vs. 0%, *p* = 0.006), and pericardial effusion (18% vs. 4%, *p* = 0.026) in a significantly higher number of SSc subjects, compared to matched controls. A positive linear correlation has been observed between disease duration and LGE progression from basal to apical left ventricular segments (*r* = 0.82, *p* < 0.001). The lower the ejection fraction, the longer the disease duration (*r* = −0.69, *p* = 0.004), and the higher the number of LGE segments (*r* = −0.78, *p* < 0.001). Multivariate analysis confirmed disease duration as independent predictor of myocardial fibrosis after adjustment for confounders (*p* < 0.001), as well as the independent association of both LGE and disease duration with impaired left ventricular ejection fraction in SSc subjects (*p* < 0.001). Doppler echocardiography showed a significantly lower E/A ratio (1.0 ± 0.3 vs. 1.3 ± 0.6, *p* = 0.002), higher E/e’ ratio (10.9 ± 2.5 vs. 6.8 ± 1.1, *p* < 0.0001) and systolic peak tricuspid regurgitation velocity (2.7 ± 0.6 m/s vs. 2.3 ± 0.5 m/s, *p* = 0.005) among SSc patients compared to controls. LGE-positive SSc subjects showed a higher E/e’ ratio compared to other SSc patients (13.5 ± 2.4 vs. 9.7 ± 1.4, *p* < 0.0001).

**Conclusions:**

These data confirm the significant prevalence of imaging findings of subtle cardiac impairment in SSc patients, thus suggesting the role of multimodality imaging by Doppler echocardiography and CMR as a potential screening approach in this subset population.

## 1 Introduction

Systemic sclerosis (SSc) is a chronic heterogeneous disorder characterized by dysregulated inflammation, widespread microvascular lesions, and fibrosis of the skin and internal organs. Although often clinically occult, cardiac impairment is common in SSc patients, with relevant prognostic implications in clinical practice (1–3). In fact, although cardiac derangement is often underestimated, it is associated with a 5-year mortality rate of 70%, with 28% of SSc patients dying due to cardiac complications (i.e., pulmonary hypertension, heart failure, and cardiac arrhythmias) (4–6). Therefore, early detection of subtle cardiac involvement in SSc subjects without clinical findings of overt cardiovascular involvement is mandatory. However, to date no single imaging tool has proven sufficiently sensitive for a comprehensive assessment of both cardiac systolic and diastolic function and myocardial tissue characterization, in order to be used as a multiparametric screening tool in this subset population. Specifically, Doppler echocardiography provides real-time detection of cardiac function and hemodynamics in clinical practice. However, despite having a good temporal resolution, its diagnostic accuracy is limited by a lower spatial resolution as well as acoustic window and user dependency compared to other imaging tools. Furthermore, although it relies largely on indirect markers of tissue health, it lacks the ability to directly assess tissue characterization (7, 8). In this regard, cardiac magnetic resonance imaging (CMR) is a useful imaging tool to complement Doppler echocardiography, due to its higher spatial resolution and lower observer variability in assessing cardiac chamber quantification and function, as well as its superior diagnostic accuracy in tissue characterization through specific techniques capable of distinguishing between different types of myocardial tissue (9, 10). Nevertheless, to date a comprehensive screening approach by multimodality imaging assessment is not a systematically adopted practice in this subset population. Our aim is to investigate the role of a multimodality imaging approach in the detection of subtle cardiac impairment in SSc patients with unremarkable clinical findings of cardiovascular impairment.

## 2 Patients and methods

### 2.1 Patients

**Figure 1:**
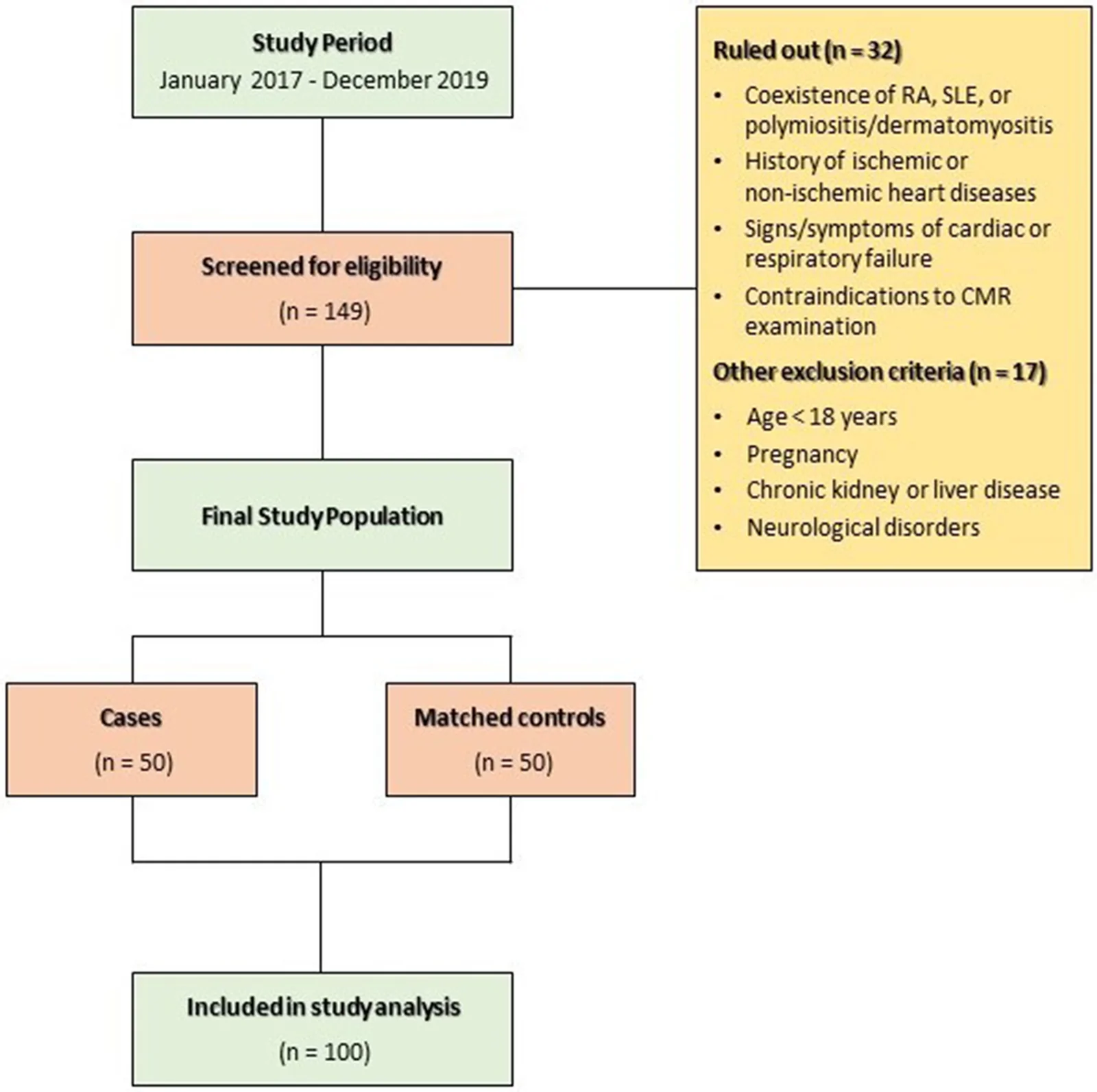
Strobe flow diagram for case-control study population recruitment and allocation. CMR, cardiac magnetic resonance; RA, rheumatoid arthritis; SLE, systemic lupus erythematosus.

This single-center case-control study was performed in SSc subjects and was conducted in full accordance with the guidelines issued by the local institutional review board. Between January 1^st^ 2017 and December 31^st^ 2019, we retrospectively collected data related to 50 consecutive unselected patients affected by SSc (mean age 53.4 ± 12.7; male/female: 5/45), diagnosed according to the criteria established by the American College of Rheumatology and the European League Against Rheumatism (11). The exclusion criteria for all SSc subjects were as follows: i) coexistence of rheumatoid arthritis, systemic lupus erythematosus, or polymyositis/dermatomyositis; ii) known history of ischemic or non-ischemic heart disease; iii) cardiac or respiratory failure; iv) contraindications to CMR examination; v) age < 18 years; vi) pregnancy; vii) chronic kidney or liver disease; and viii) neurological disorders (Figure 1). Among the enrolled subjects, 27 showed limited SSc and 23 diffuse SSc according to the LeRoy classification (12). The mean disease duration was 6.3 ± 1.9 years. Disease duration was assumed to start from the appearance of Raynaud's phenomenon. All patients underwent Doppler echocardiography within one month before or after CMR. Physical examination, 12-lead ECGs, chest x-rays, and biochemical tests, including antibody screening were performed on all patients. Demographic data and anthropometric measurements of weight and height were provided, in order to calculate body mass index (BMI, Kg/m^2^) and body surface area (BSA, m^2^). None of the SSc patients had any history of cardiac disease or heart complaints, and physical examination was unremarkable. 12-lead ECG examination revealed sinus tachycardia in 4 (8%) SSc subjects and nonspecific repolarization abnormalities in 7 (14%) subjects. None of the ECG examinations showed any remarkable rhythm or morphology abnormality. Biochemical examinations detected anti-nuclear antibodies in 46 (92%) SSc patients and antibodies to extracellular nuclear antigens in 37 (74%) patients, while no cases showed positivity to both antibodies.

### 2.2 Cardiac magnetic resonance imaging

CMR was carried out with a 1.5 Tesla magnetic resonance imaging scanner (Intera, Philips Medical Systems, Best, The Netherlands) using a 32-channel phased-array cardiac coil for signal reception. Cardiac synchronization was obtained with four nonmagnetic electrodes placed on the left anterior hemithorax and connected to the console using optical fibers. Electrodes were placed on the chest in order to maximize the R-wave triggering. Cine sequences were obtained at baseline with segmented cardiac-gated multiphase steady-state free-precession (SSFP) sequences with breath hold in four axes (short-axis, long-axis, four-chamber, and two-chamber views). Imaging parameters were as follows: repetition time (TR) 3.1–4 ms, echo time (TE) 1.5–2 ms, flip angle (FA) 60°, slice thickness 8 mm, interslice gap 2 mm, image matrix 256 × 192, field of view (FOV) 380-430 mm, SENSE factor 2 in the phase-encoding direction, multiphase (16–25 phases/R-R interval). For the assessment of myocardial edema, fat-suppressed T2-weightened turbo spin echo sequences (TR 2000ms, TE 80 ms, FA 90°, 2 acquisitions, slice thickness 8 mm, interslice gap 10%, image matrix 176 × 256, FOV 380-400 mm, SENSE factor 2 in phase-encoding direction) were collected in the four-chamber and the short-axis views. Delayed contrast enhancement sequences were acquired in short-axis and long-axis views 12 to 15 min after intravenous administration (0.2 mmol/Kg) of gadolinium chelate (Bayer Schering Pharma, Berlin, Germany), with an inversion-recovery 2-dimensional turbo gradient-echo sequence cardiac-triggered in diastole (TR 3.5–5.2 ms, TE 1.1–1.5 ms, FA 15°, turbo factor 40–50, 10–14 slices, slice thickness 8–12 mm, interslice gap 4–6 mm, image matrix 125 × 256 to 272 × 304, FOV 330–430 mm). The left ventricle (LV) was divided into 17 segments according to the American Heart Association standardized myocardial segmentation (13). For each segment, regional wall motion was visually defined as normal, hypokinetic, akinetic, or dyskinetic. End-diastolic and end-systolic volumes were calculated for both ventricles by defining the endocardial contour of end-diastolic and end-systolic short-axis cine images. The volume of single slices was subsequently summed to calculate volumes in end-diastole and end-systole. All volumetric values were indexed to BSA to account for differences in body size. Right ventricular (RV) hypertrophy was defined by a thickness ≥ 5 mm. Myocardial edema was defined assessing the presence of increased intramyocardial signal intensity on T2-weighted images. Delayed contrast enhancement was defined as an area meeting all of the following criteria: a signal intensity value > 2 SD above the mean of normal myocardium; presence in the same myocardial segment in at least two different planes; and presence in identical planes on two different acquisitions with the appropriate inversion time. Delayed contrast enhancement was visually assessed for each myocardial segment; its extent was classified as subendocardial, mid-myocardial, and transmural, while its pattern was defined as focal, linear or patchy. Pericardial effusion was virtually assessed on the cine and axial HASTE images. CMR abnormalities were independently assessed by two operators (SS and CB) blinded to the clinical findings. In case of discrepancy a consensus was reached by discussion. No contraindications for CMR or the use of intravenous contrast medium were observed in any of the patients, according to the current international guidelines.

### 2.3 Doppler echocardiography

All patients underwent Doppler echocardiography (GE-Vigmed Vivid 7 system (GE Medical Systems, Milwaukee, USA), within one month before or after CMR. Echocardiographic examinations were performed using a commercially available equipment with a phased array system (HP) with a 2–4 MHz transducer. Patients were examined in the left lateral recumbent position, with the transducer positioned along the left sternal border. Diastolic function was assessed through pulsed-wave (PW) mitral inflow Doppler and tissue Doppler imaging (TDI). Mitral inflow velocities were recorded in 4-chamber views using PW Doppler. with the sample volume placed at the level of the mitral valve leaflet tips. The following diastolic parameters were measured: early (E) and atrial (A) peak mitral inflow velocities, E/A ratio, deceleration time (DT), and isovolumetric relaxation time (IVRT), measured from aortic valve closure to mitral valve opening. DT is defined as the time needed for peak E to decline to its baseline and IVRT as the time from aortic valve closure to the onset of LV inflow. TDI was performed in the apical 4-chamber view by positioning the sample volume at the septal and lateral borders of the mitral annulus. LV outflow tract velocity time integral (LVOT VTI) was measured by placing a PW sample volume in the apical 5-chamber or 3-chamber view by positioning the gate just proximal to the aortic valve in order to capture the laminar blood flow and trace the velocity envelope. Colour Doppler tricuspid flow pattern was also measured to calculate RV filling. In case of detectable tricuspid regurgitation, continuous-wave (CW) Doppler echocardiography was used to measure the peak systolic pressure gradient across the tricuspid valve. Digital lops, obtained with the patient in the left lateral decubitus position, were captured and then analysed using dedicated off-line software (EchoPAC 13:0; GE Medical Systems, Milwakee, USA). The tracings were collected and read independently by 2 echocardiographers (RS and GMR) with intra-observer and inter-observer variability of less than 1% and 4.5%, respectively.

### 2.4 Control subjects

Fifty controls, matched for age, sex and cardiovascular risk predictors who underwent the same CMR and Doppler echocardiography examinations were selected for comparison. At the baseline, they were all free from any cardiovascular history and had unremarkable clinical findings, 12-lead ECGs and chest x-rays (Table 1).

**Table 1:** Baseline characteristics of the study population.

Variable	SSc subjects (*n* = 50)	Controls (*n* = 50)	*P*
Age (year)	53.4 ± 12.7	58.8 ± 13.1	0.384
Male/Female (*n*)	5/45	9/41	0.249
BMI (kg/m^2^)	23.7 ± 3.1	24.1 ± 3.1	0.484
BSA (m^2^)	1.7 ± 0.1	1.7 ± 0.2	0.340
Arterial hypertension (*n*, %)	9 (18)	13 (26)	0.932
Smoking habits (*n*, %)	11 (22)	16 (32)	0.260
Dyslipidemia (*n*, %)	12 (24)	7 (14)	0.203
Diabetes mellitus (*n*, %)	2 (4)	3 (6)	0.646
Alpha-blocker (*n*, %)	2 (4)	4 (8)	0.400
Beta-blocker (*n*, %)	1 (2)	2 (4)	0.558
ACEI/ARB (*n*, %)	3 (6)	5 (10)	0.461
Thiazide (*n*, %)	1 (2)	3 (6)	0.307
CCB (*n*, %)	2 (4)	3 (6)	0.646

ACEI, angiotensin converting enzyme-inhibitor; ARB, angiotensin receptor blocker; BMI, body mass index; BSA, body surface area; CCB, calcium channel blocker; SSc, systemic sclerosis.

### 2.5 Statistical analysis

Statistical analysis was carried out using the Statistica 13.1 software for Windows (StatSoft, Inc., Tulsa, Oklahoma). Quantitative variables were expressed either as numbers (percentage of total) or mean ± standard deviation. The statistical significance of the results from each of the two groups was determined by means of either chi-square test or t-test, as appropriate. Correlations between numerical parameters were assessed using Pearson's correlation and a multivariate analysis using a logistic regression model. A *p* value < 0.05 was considered statistically significant.

## 3 Results

**Figure 2:**
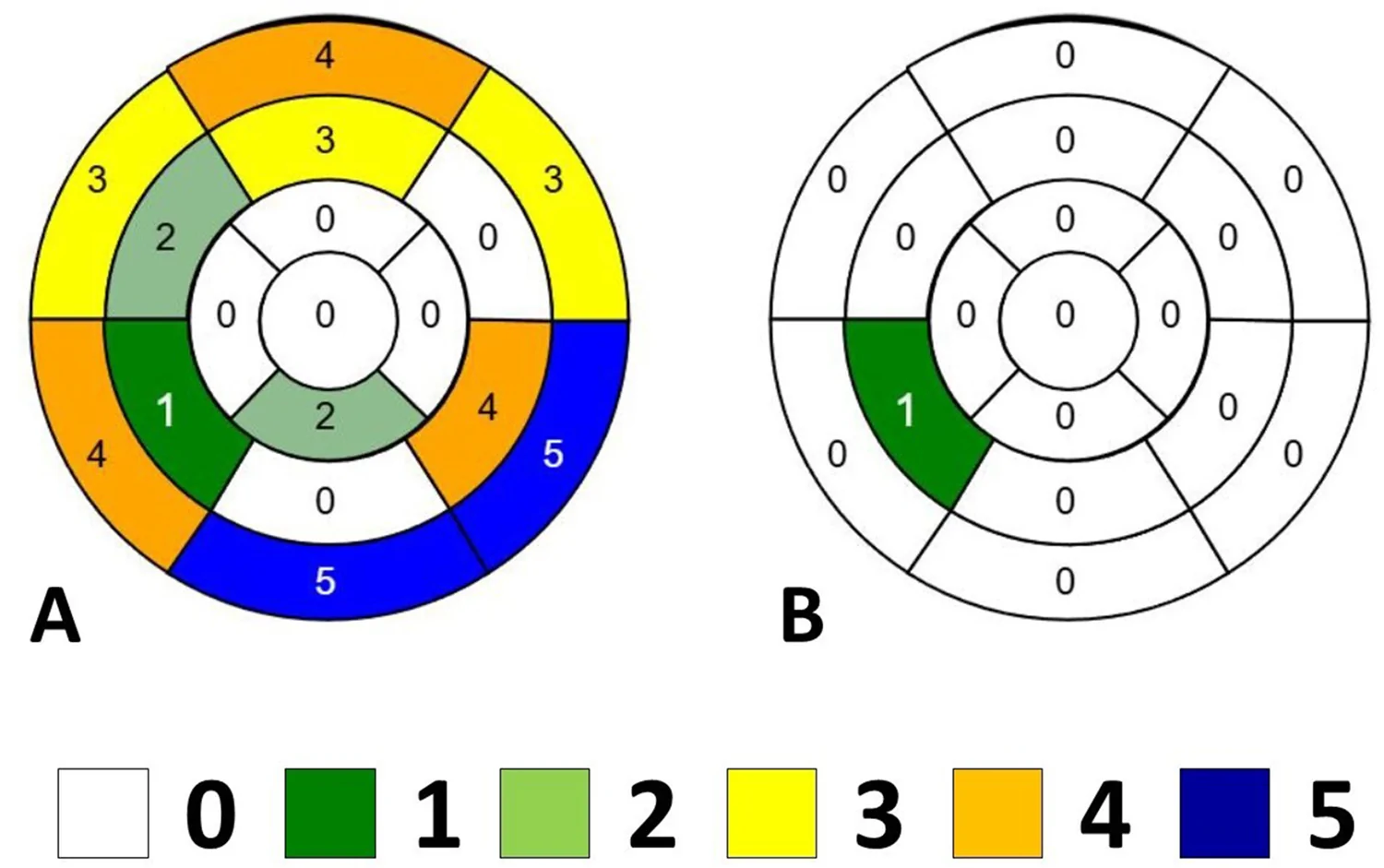
Distribution of late gadolinium enhancement (LGE) to the segments of the left ventricle accordingly to the American Heart Association standardized myocardial segmentation among SSc subjects **(A)** and matched controls **(B)** data expressed as observed numbers of LGE positive segments.

Among SSc patients CMR showed a significant increase in indexed ventricular volumes, left atrial end-systolic volume and LV mass as well as a lower RV ejection fraction, compared to the matched controls (Table 2). LV myocardial late gadolinium enhancement (LGE) was detected in 15 (30%) SSc patients vs. 1 (2%) controls (*p* = 0.0001). All LGE-positive SSc patients showed a non-ischemic, mid-myocardial linear pattern, following a non-coronary distribution. A base-to-apex LGE progression gradient was detected among SSc subjects (Figure 2). Specifically, all SSc patients with detectable LGE invariably showed the involvement of LV basal segments, and its detection was significantly higher than in patients with either mid-ventricular (100% vs. 60%, *p* = 0.006) or apical involvement (100% vs. 13.3%, *p* < 0.0001). Similarly, the number of SSc patients with LGE in the mid-ventricular segments was greater than those with LGE in the apical segments (60% vs. 13.3%, *p* = 0.008). SSc patients with LV LGE had a significantly longer disease duration compared to those without LGE (8.4 ± 0.9 vs. 6.1 ± 0.2 years, *p* < 0.0001). We found a good positive linear correlation between disease duration and LGE ventricular segments following a disease progression from basal to apical segments (*r* = 0.82, *p* < 0.001). Additionally, a significantly lower LV ejection fraction was detected among LV LGE-positive SSc patients, compared to those without LGE (49.0 ± 8.6% vs. 69.6 ± 1.1%, *p* < 0.0001). Concerning the duration of SSc, we found that the lower the ejection fraction, the longer the disease duration (*r* = −0.69, *p* = 0.004), and the higher the number of LGE segments (*r* = −0.78, *p* < 0.001). The independent association between disease duration and LGE development persisted after adjustment for age, sex, arterial hypertension smoking habits, diabetes mellitus, dyslipidemia, and disease subtypes in a multivariate regression model (*R*^2^ = 0.54, *F* (8,41) = 6.12, *p* < 0.001). Additionally, multivariate logistic regression analysis confirmed that LGE itself and disease duration are both independent predictor of lower ejection fraction after adjustment for confounders (*R*^2^ = 0.81, *F* (9,40) = 18.81, *p* < 0.001). Moreover, 11 SSc patients with positive LV LGE also showed focal enhancement at the anterior and/or posterior RV insertion sites, compared to 3 matched controls (22% vs. 6%, *p* = 0.021). Among these patients, CMR showed a lower RV ejection fraction compared to other SSc patients (40.9 ± 7.2% vs. 53.7 ± 8.2%, *p* < 0.0001). In this subset group, concurrent hypokinesia of the RV free wall was detected in 6SSc patients, compared to no wall motion abnormalities observed in the controls (*p* = 0.012). T2-weighted CMR sequences showed myocardial edema in 7 (14%) SSc patients and none of the controls (*p* = 0.006). All of these subjects had no clinical or biochemical findings suggestive of myocarditis and showed a concurrent delayed enhancement in the same ventricular segments. Pericardial effusion was observed in 9 (18%) SSc patients and 2 (4%) controls (*p* = 0.026). It was of mild size (space between the two pericardial layers ≤ 10 mm at end-diastole) in all the observed subjects, with no associated hemodynamic impairment. Compared to the matched controls, in SSc patients mitral PW Doppler analysis showed higher values of peak A-wave (*p* < 0.0001), a lower E/A ratio (*p* = 0.002) and prolonged DT (*p* < 0.0001). Inversion of E/A ratio was noticed in 25 (50%) SSc patients and 12 (24%) controls (*p* = 0.007). Mitral tissue Doppler imaging analysis showed a lower trans-mitral early filling peak velocity e’ (*p* < 0.0001) and higher E/e’ ratio among SSc patients compared to the controls (*p* < 0.0001) (Table 3). A lower peak e’-wave (6.8 ± 1.2 cm/s vs. 7.8 ± 0.9 cm/s, *p* = 0.029) and higher E/e’ ratio (13.5 ± 2.4 vs. 9.7 ± 1.4, *p* < 0.0001) were found among LGE-positive SSc patients, compared to other SSc subjects. Tricuspid CW Doppler analysis showed a higher systolic peak regurgitation velocity among SSc patients, compared to the controls (2.7 ± 0.6 m/s vs. 2.3 ± 0.5 m/s, *p* = 0.005). A peak regurgitation velocity > 2.8 m/s was detected in 17 (34%) SSc subjects and 5 (10%) controls (*p* = 0.004). No significant correlation was found between estimated pulmonary artery systolic pressure and RV fibrosis, RV volumes or ejection fraction.

**Table 2:** Cardiac magnetic resonance findings of the study population.

Variable	SSc subjects (*n* = 50)	Controls (*n* = 50)	*P*
LV indexed EDV (mL/m^2^)	75.2 ± 14.1	55.6 ± 10.1	< 0.0001
LV indexed ESV (mL/m^2^)	29.0 ± 9.5	22.7 ± 6.1	0.0001
LV ejection fraction (%)	59.5 ± 7.1	62.1 ± 8.1	0.093
LV mass (g/m^2^)	84.1 ± 17.6	65.8 ± 12.3	< 0.0001
LA indexed ESV (mL/m^2^)	27.9 ± 6.3	24.7 ± 6.0	0.012
RV indexed EDV (mL/m^2^)	69.0 ± 13.4	51.2 ± 8.8	< 0.0001
RV indexed ESV (mL/m^2^)	34.0 ± 8.7	23.1 ± 5.8	< 0.0001
RV ejection fraction (%)	50.9 ± 7.8	57.3 ± 6.9	< 0.0001
RV free wall thickness (mm)	4.0 ± 1.4	3.6 ± 1.2	0.128
RA indexed ESV (mL/m^2^)	21.2 ± 4.2	20.3 ± 4.4	0.339
Myocardial LGE (*n*, %)	15 (30%)	4 (8%)	0.005
Myocardial edema (*n*, %)	7 (14%)	0 (0%)	0.006
Pericardial effusion (*n*, %)	9 (18%)	2 (4%)	0.026

EDV, end-diastolic volume; ESV, end-systolic volume; LGE, late gadolinium enhancement; LA, left atrium; LV, left ventricle, RA, right atrium; RV, right ventricle.

**Table 3:** Doppler echocardiography findings of the study population.

Variable	SSc subjects (*n* = 50)	Controls (*n* = 50)	*P*
Peak E-wave (cm/s)	75.4 ± 16.0	72.2 ± 18.0	0.350
Peak A-wave (cm/s)	80.2 ± 20.2	58.9 ± 12.7	< 0.0001
Mitral E/A ratio	1.0 ± 0.3	1.3 ± 0.6	0.002
Isovolumic relaxation time (ms)	74.6 ± 14.2	73.5 ± 12.8	0.696
Deceleration time (ms)	230.8 ± 47.1	197.9 ± 26.9	< 0.0001
Peak e’-wave (cm/s)	7.5 ± 1.5	11.0 ± 2.2	< 0.0001
Mitral E/e’ ratio	10.9 ± 2.5	6.8 ± 1.1	< 0.0001
LVOT VTI (cm)	19.5 ± 2.8	19.3 ± 4.0	0.782
Systolic peak TR velocity (m/s)	2.7 ± 0.6	2.3 ± 0.5	0.005
S’ tricuspid annular velocity (cm/s)	10.5 ± 1.4	11.4 ± 1.9	0.002

LVOT VTI, left ventricle outflow tract velocity time integral; TR, tricuspid regurgitation.

## 4 Discussion

To the best of our knowledge, this is the largest comprehensive case-control study examining the role of a multimodality imaging screening approach using CMR and Doppler echocardiography to investigate subclinical cardiac impairment in SSc subjects with unremarkable cardiovascular objectivity. CMR enabled us to investigate an early phenotypic involvement of subtle cardiac impairment in SSc patients due to its greater reproducibility in volumetric quantification and greater accuracy in myocardial tissue characterization, compared to cardiac ultrasound (14–16). Specifically, CMR is considered the best imaging tool for detecting myocardial fibrosis, a pathophysiologic hallmark of cardiac involvement in SSc, strongly influencing prognostic outcomes in this subset population (17, 18). In this scenario, LGE was detected in 15% of our SSc population, in line with previous investigations by Nassenstein et al. (19). Conversely, data from Tzelepis et al. found LGE in more than 60% of their collectives (20). These discrepancies can be partially explained by a shorter disease duration (respectively 6.3 ± 1.9 vs. 9.9 ± 7.5 years) and a different subset population compared to our investigation (which only involved SSc subjects without overt cardiovascular impairment). These findings reinforce the evidence concerning the progression of myocardial fibrosis at more advanced stages of the disease. This hypothesis further supported by evidence of a base-to-apex progression of LV myocardial fibrosis in SSc subjects over time, and by a significant association between the progression of fibrosis to more distal LV segments and a longer disease duration, in parallel with a progressive impairment of systolic function. This distinctive progression pattern of myocardial disruption in SSc seems to be sustained by both a microvascular functional trigger (i.e., a higher oxygen demand from LV basal segments and a thinner capillary bed, which is more susceptible to repeated ischemic-reperfusion injuries) and a mechanical wall stress component, as the LV basal segments undergo significantly more longitudinal mechanical strain and torsion during the cardiac cycle compared to the LV apical segments, which creates a profibrotic environment (10, 21). Taken together, these findings suggest a progressive structural and functional myocardial deterioration over time, consistently with the natural history of heart involvement in SSc. In line with previously reported data, nearly 20% of our SSc patients showed focal myocardial fibrosis at the RV insertion points and were associated with a lower RV ejection fraction and longer disease duration (18). The presence of myocardial fibrosis at the RV hinge points is common in patients with pulmonary hypertension (either with or without SSc) (22, 23). However, we did not find a close relationship between increased pulmonary arterial systolic pressure and RV insertional fibrosis in our subset population. Notably, all patients showed concurrent delayed enhancement in the LV segments, and over half of them exhibited concurrent hypokinesia of the RV free wall. Furthermore, remarkably the RV free wall can also be affected by replacement fibrosis. However, its detection is often challenging and may be underestimated due to its peculiar anatomical features (thin and trabeculated) (10). Additionally, RV and/or LV ejection fraction was altered in almost one third of SSc patients despite the mean values remaining within normal ranges, with no evidence of overt cardiac failure. Taken together, these findings suggest that LV and/or RV systolic impairment is likely a direct consequence of myocardial fibrosis, and reflects a global structural replacement process that progresses over time, as preciously suggested by other authors (17, 24, 25). The main pathogenic mechanism of cardiac involvement in SSc is still debated. A first hypothesis proposed by Bulkey et al. suggests that myocardial damage and replacement fibrosis follow recurrent ischemic events and reperfusion injuries caused by intermittent intramyocardial arterial spasm (also named ‘myocardial Raynaud's phenomenon’) (26). However, this hypothesis is mainly supported by radionuclide data obtained using non-gated thallium single-photon emission computed tomography, which is well known for a high number of false positive results (27, 28). In this perspective, the consistently non-ischemic pattern of myocardial fibrosis observed in our SSc population - which spares the subendocardial layer and is unrelated to any specific coronary distribution - suggests alternative pathogenic pathways other than an ischemic trigger, thus supporting the theory of an underlying immune-mediated process surrounding the pathogenic mechanism of cardiac involvement in SSc (29–31). In this scenario, the detection of myocardial edema (indentified as hyperintense areas on T2-weighted images) following a non-coronary distribution pathway is also highly suggestive of a concurrent inflammatory aetiology. The prevalence of myocardial edema in SSc is variously reported in the literature. It can be detected simultaneously or before the appearance of LGE, as immune dysregulation in SSc leads to leukocyte diapedesis, upregulation of adhesion molecules and the release of cytokines, with a consequent increase in the distribution spaces of gadolinium chelates (10, 20, 32). In line with previous investigations, in our study all SSc patients with identified myocardial edema showed delayed enhancement in the same ventricular segments without any coronary correlation (17, 18). Moreover, in our study population mild size pericardial effusion was discovered in a significant number of SSc patients compared to controls. Pericardial effusion in SSc is generally clinically silent and most cases has no prognostic significance (9, 10, 17). Unlike other immune-mediated disorders (such as systemic lupus erythematosus or rheumatoid arthritis) in which the primary mechanisms seem related to acute, intense serositis triggered by immunocomplex and auto-antibody deposition in the pericardial tissue, the pathogenesis of pericardial effusion in SSc is thought to be driven by microvascular damage and fibrosis, leading to chronic, progressive pericardial fluid accumulation. These distinct pathogenic mechanisms are supported by a general lack of response of pericardial disease to corticosteroid treatment in SSc compared to other autoimmune diseases (1, 33, 34). To date, CMR is not currently recommended as part of the baseline routine assessment for all patients diagnosed with SSc, and there is no universal consensus on routine timeline mandating when to perform a CMR in SSc subjects, while it is instead suggested when standard screening tools (i.e., 12-lead ECG, Doppler echocardiography or cardiac biomarkers) reveal subclinical abnormalities. However, as MRI findings of primary heart involvement have been shown to be detected even before clinical manifestation or the development of subclinical abnormalities at standard screening tools, a growing consensus highlights the use of CMR within a multiparametric preclinical work-up in all SSc subjects at different points of their medical journey (35). A broad adaptation of this approach would require a cost-effectiveness validation and further longitudinal studies in order to provide evidence on whether a multiparametric screening approach involving CMR could significantly impact on clinical perspective and lead to improved outcomes in SSc patients. On the other hand, Doppler echocardiography plays a pivotal role in the non-invasive assessment of hemodynamics in clinical practice, as well as being the primary tool for assessing diastolic function, which is often compromised in SSc (36–38). However, the underlying mechanisms surrounding diastolic impairment in SSc are controversial. Aguglia et al. suggests that LV diastolic dysfunction is only seen in patients with predisposing conditions potentially affecting LV diastolic function (i.e., aging, systemic or pulmonary hypertension, diabetes mellitus, obesity, valvular heart disease, or coronary artery disease) (39). Conversely, other authors suggest that diastolic dysfunction in SSc may reflect primary myocardial fibrous tissue replacement, irrespective of other predisposing conditions (5, 40, 41). In our study, mitral inflow Doppler assessment showed that LV diastolic abnormalities are unrelated to the aforementioned risk predictors in SSc subjects. Furthermore, we observed that among SSc patients the concurrence of myocardial fibrosis was significantly associated with both an impaired longitudinal mitral annular velocity during early diastole and a higher LV filling pressure, while we did not find any significant correlation between diastolic dysfunction and disease duration. Taken together, our findings reinforce the hypothesis that LV diastolic dysfunction in SSc patients may be mainly driven by myocardial fibrous tissue deposition, rather than by other conventional factors leading to diastolic impairment in the general population (10, 42). Using CW Doppler analysis of tricuspid regurgitation, we observed a significantly higher estimated pulmonary artery systolic pressure among SSc subjects compared to the matched controls, despite mean values remaining within physiological ranges. Notably, in our study population increased pulmonary artery systolic pressures were rather mild, which likely explains the lack of correlation with RV dilatation and/or dysfunction and RV free wall hypertrophy. Consistently with previous investigations, we found no significant differences in cardiac imaging findings between limited and diffuse SSc patients (17–19). The considerable differences in the detection rate of myocardial fibrosis in the present study compared to autoptic data (where myocardial fibrosis is reported in up to 70% of SSc subjects) can be mainly explained by differences in the study population and the much higher spatial resolution of histology compared to *in-vivo* CMR (17, 19, 43). Recent advances in artificial intelligence and radiomics are looking to potentially overcome some of the limitations related to technical accuracy and interpretation in cardiovascular imaging, in order to improve accuracy for risk stratification, diagnosis, and follow-up of patients with subclinical findings of cardiovascular impairment. However, despite such advances, to date they are still scarcely routinely applied in clinical practice due to the absence of standardized parameters of acquisition and lack of external validation (44, 45).

## 5 Limitations

We acknowledge that our study carries several limitations. First, our imaging findings are not supported by histological confirmation through myocardial biopsies, since this procedure was judged to be too invasive to be incorporated into the study of an asymptomatic population. In addition, although effective in identifying ischemic scar areas, biopsies performed in multiple subendocardial regions would most likely prove inconclusive for the detection of non-ischemic mid-myocardial lesions. Therefore, in certain cases, such confirmations would have to come from necroscopic studies. Secondly, although coronary artery disease seems unlikely in our detected LGE-positive patterns, it cannot be conclusively ruled out as selective coronary angiography has not been performed in our study population. Additionally, T1 mapping, which can be used to calculate and map the distribution of the myocardial extracellular volume fraction, was not available at the time of this study. Specifically, parametric mapping techniques like native T1 and extracellular volume provide an accurate assessment of tissue relaxation times, enhancing diagnostic sensitivity for detecting diffuse interstitial fibrosis, which can be the only abnormal parameter in SSc subjects and whose detection can be missed when relying only on LGE. Furthermore, despite collected data are limited by the small sample size, the study was adequately powered for subgroup analysis. Additionally, a carefully matched control group strengthened the design of our study by pairing SSc subjects and controls on the basis of shared characteristics., thus reducing bias and minimizing variance between the two groups. Furthermore, strain analysis software was not available at the time of this study. Nevertheless, the use of CMR in the study population made it possible to overcome inherent limitations related to poor image quality, out-of-plane motion, segmental variability, and geometric artifacts (46). Finally, our study did not supply findings about longitudinal outcomes and was not able to provide the prognostic significance of cardiac abnormalities detected by imaging tools over time, due to the lack of a follow-up period.

## 6 Conclusions

Subtle cardiac involvement is common in patients with SSc, and encompasses both structural and functional impairment, which progresses over time. Our study suggests the role of multimodality imaging by Doppler echocardiography and CMR as a potential screening approach in cardiac asymptomatic subjects with SSc, in order to noninvasively define the pattern and extension of myocardial fibrosis and its relationship with both systolic and/or diastolic ventricular dysfunction in this subset population before the appearance of clinically overt heart disease. Further longitudinal studies are advisable in order to investigate the natural history of the disease and the progression of cardiovascular impairment in the long-term follow-up.

## Data Availability

The original contributions presented in the study are included in the article/Supplementary Material, further inquiries can be directed to the corresponding author.
